# Estimating T-cell repertoire diversity: limitations of classical estimators and a new approach

**DOI:** 10.1098/rstb.2014.0291

**Published:** 2015-08-19

**Authors:** Daniel J. Laydon, Charles R. M. Bangham, Becca Asquith

**Affiliations:** Section of Immunology, Wright-Fleming Institute, Imperial College School of Medicine, London W2 1PG, UK

**Keywords:** T-cell receptor repertoire, diversity, species richness

## Abstract

A highly diverse T-cell receptor (TCR) repertoire is a fundamental property of an effective immune system, and is associated with efficient control of viral infections and other pathogens. However, direct measurement of total TCR diversity is impossible. The diversity is high and the frequency distribution of individual TCRs is heavily skewed; the diversity therefore cannot be captured in a blood sample. Consequently, estimators of the total number of TCR clonotypes that are present in the individual, in addition to those observed, are essential. This is analogous to the ‘unseen species problem’ in ecology. We review the diversity (species richness) estimators that have been applied to T-cell repertoires and the methods used to validate these estimators. We show that existing approaches have significant shortcomings, and frequently underestimate true TCR diversity. We highlight our recently developed estimator, DivE, which can accurately estimate diversity across a range of immunological and biological systems.

## 1. Introduction

The human T-cell receptor (TCR) repertoire—the range of different TCRs expressed—plays a vital role in host defence. By recombination, random insertion, deletion and substitution, the small set of genes that encode the T-cell receptor has the potential to create between 10^15^ and 10^20^ TCR clonotypes (a clonotype is a population of T cells that carry an identical TCR) [1,2]. However, the actual diversity of a person's TCR repertoire cannot possibly lie in this range. There are only an estimated 10^13^ cells in the human body [3], and many clonotypes are of high abundance due to strong selection forces (for example, thymic education or antigen specificity). The actual, or realized, diversity of the human TCR repertoire remains unknown. The term ‘diversity’ is commonly used to mean either the number of classes (also known as ‘species richness'), or the degree of dispersion among those classes. In this study, we use the term ‘species' to refer to a single TCR clonotype, and ‘diversity’ to refer to the number of TCR clonotypes.

**Figure 1.**
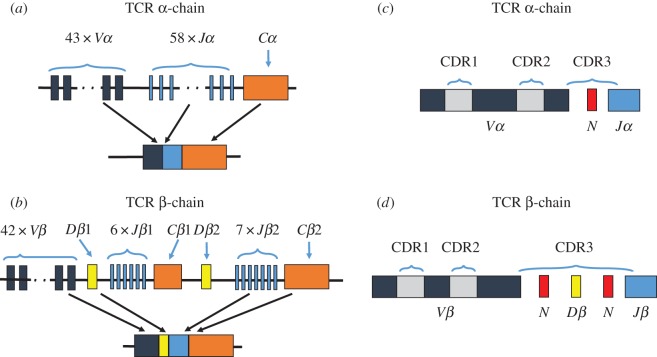
T-cell receptor gene rearrangement. (*a*) Variable (*V*), joining (*J*) and constant regions (*C*) constitute the TCR α-chain. (*b*) Variable (*V*), joining (*J*) and constant regions (*C*) constitute the TCR β-chain, with an additional diversity (*D*) region. Segments from each region are recombined, with additional nucleotide additions, to generate each rearranged TCR. These processes generate substantial T cell diversity. (*c,d*) Hypervariable complementarity-determining regions (CDR1-CDR3) of the α-chain (*c*) and β-chain (*d*). CDR1 and CDR2 regions are encoded on the *V* region, while the most variable CDR3 region straddles the *V*(*D*)*J* junction.

TCRs are heterodimers and fall into two classes: TCR-αβ and TCR-γδ; γδ T cells constitute 1–10% of the T-cell repertoire [4]. A variable (*V*), joining (*J*) and constant region (*C*) constitute the TCR α- and γ-chains. The TCR β- and δ-chains are also made up of a *V*, *J* and *C* region, with an additional diversity (*D*) region [5]. One segment from each region is recombined, with additional nucleotide additions and/or deletions, to generate each rearranged TCR (figure 1). This recombination generates high T-cell diversity [1] and enables the recognition of millions of antigens [6]. While *V*(*D*)*J* gene rearrangement is believed to be random [7], some clonotypes are produced more commonly than others [2,8], leading to unequal frequencies of naive T-cell clonotypes and to ‘public’ clonotypes, i.e. clonotypes shared between people. This unequal frequency distribution is believed to be due to a process known as convergent recombination, whereby certain nucleotide sequences can be produced using a greater variety of recombination events; certain amino acid sequences can be made by a greater number of nucleotide triplets; and certain TCRs require fewer nucleotide insertions, deletions or substitutions [9].

The third complementarity-determining region 3 (CDR3) of both the TCR α- and TCR β-chains straddles the *V*(*D*)*J* junction [10,11] (figure 1*b*), the primary site of antigen contact [5]. The CDR3 is the region most affected by recombination [12], and the CDR3 region of the β-chain accounts for most of the variation within a person's T-cell repertoire. Antigenic cross-reactivity of T cells results in a discrepancy between structural diversity (the number of different nucleotide or amino acid TCR combinations in the host) and functional diversity (the number of different antigens recognized by the T-cell repertoire) [1].

## 2. Why is T-cell receptor diversity important?

TCR diversity is associated with the effective control of viral infections and other pathogens [13–15]. The number of clonotypes observed in the blood in one person has been reported to decrease with age [16–19], viral challenge [15,20,21], immunization [22] and as a result of immune suppression, for example after haematopoietic stem cell transplantation (HSCT) [23]. TCR diversity has also been positively associated with autoimmunity in both mice [24,25] and humans [26]. Accurate quantification of diversity is important to assess the extent of immune convergence (sharing of clonotypes between people) [7,24,27–29].

Species diversity is also important in many systems outside T-cell immunology, for example, in estimating the repertoire of antibody classes [30,31], assessing the size of the metagenome in microbial communities [32,33] and measuring the rate of evolution of quasi-species of a pathogenic virus [34]. The original motivation for estimating diversity comes from population ecology, where the question of how many species there are in a given population gives rise to the ‘unseen species problem’: how many species are present, but unobserved, in the population of interest? Typically, there is a nonlinear relationship between the number of individuals (e.g. a T cell, a microbe) and the number of ‘species' (e.g. a clonotype or viral variant), and so diversity cannot usually be estimated through linear scaling.

## 3. Why is estimating diversity difficult?

Estimating the diversity of the T-cell repertoire is difficult for many reasons. First, the repertoire is highly diverse. Given the number of T cells, (assumed to be of the order of 10^12^ [35,36]), a diversity of (say) 10^7^ clonotypes [36] is unlikely to be directly observed owing to the limited volume of blood that can be taken from a person at any one time, and to the heavy-tailed frequency distributions with highly non-uniform clonotype abundances [19,37].

Second, the precise relationship between the diversity of different TCR-α and TCR-β sequences and the actual TCR functional diversity is unclear. Most recent studies focus on the CDR3 region [5,24,38–40], because it is the most variable region and because it is short enough to be captured in a single sequence read [30]. However, a T-cell receptor consists of pairings between either α and β chains or γ and δ chains; this adds a further level of diversity that is not routinely captured by many sequencing approaches. Furthermore, the relationship between TCR sequence and three-dimensional structural diversity and functional diversity are not fully understood [40,41].

Third, laboratory techniques that give absolute and unbiased estimates of clonotype frequency are technically challenging. Early studies measured TCR diversity qualitatively, where different clonotypes were identified visually as discrete bands on genomic southern blots [42–44]. Other approaches [45] used flow cytometry to measure the average observed frequency of each clonotype, reasoning that if this frequency was low then the population was more diverse.

Greater precision was achieved with spectratyping [22,46,47], where the number of different CDR3 lengths is used as a proxy for the number of clonotypes. The degree to which the frequency distribution of CDR3 lengths deviates from normality is used as a metric of clonal expansion and thus of reduced diversity (because of limited lymphocyte capacity) [21,26]. Although this inference seems reasonable, expansion of some clonotypes does not imply the extinction of other clonotypes, merely their reduced relative frequency. Spectratyping produces incomplete sequence information [10] without further subcloning of the PCR product [2,48,49] which is low-throughput and labour-intensive [26,40].

High-throughput sequencing (HTS) allows greater sequencing depth and significantly more accurate quantification of TCR clonotype abundance [39], albeit at a greater expense than spectratyping [10]. However, HTS is still subject to PCR bias and sequencing error, with the consequences that clonotype abundances can be drastically distorted and that non-existent clonotypes can be recorded, thus falsely increasing the observed diversity [50].

## 4. Unbiased sequencing of T-cell receptor diversity is insufficient for diversity estimation

5′ rapid amplification of cDNA ends (RACE) is reported to suffer from markedly less bias than other HTS approaches [5,51]. Nevertheless, 5′ RACE (and unbiased sequencing more generally) is unlikely to be sufficient for diversity estimation. Diversity estimation usually makes use of two quantities: the relative abundances of observed species, and the extent to which each species is repeatedly observed in the sample. If PCR amplification is unbiased, then relative abundances will be preserved but the degree of repetition in the sample will not.

## 5. ‘Exhaustive sequencing’ cannot capture full repertoire diversity

Because all T cells within a sample of blood will not usually be detected in a single sequencing experiment, many researchers have used ‘exhaustive sequencing’ [37,38,52], i.e. the library is sequenced with the greatest possible depth, to maximize the number of reads per clonotype. It can then be justifiably concluded that further sequencing of the same library would not yield greater observed diversity. It is therefore tempting to conclude that the sample of blood contains a complete census of clonotypes in the periphery. However, such a conclusion would be false.

**Figure 2.**
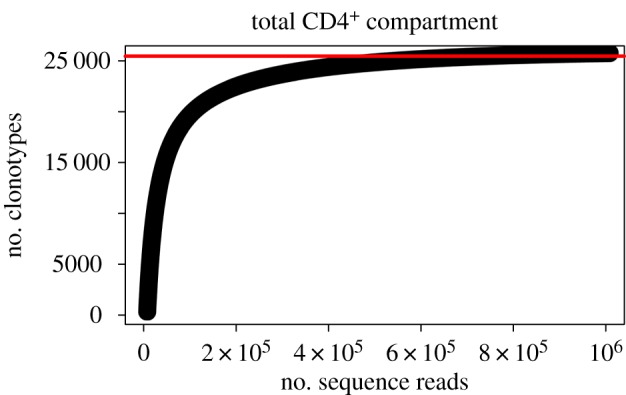
PCR amplification can lead to ‘false saturation’ of rarefaction curves. Example of ‘exhaustive sequencing’ of CD4^+^ T cell compartment in a healthy donor. Unbiased sequence data was obtained through 5′ rapid amplification of cDNA ends (RACE) [53]. The rarefaction curve approaches saturation, falsely implying that further sequencing would not yield many additional clonotypes. However, the approximate saturation value of 2.5 × 10^4^ is not a realistic estimate of total CD4^+^ TCR diversity. For example, Robins *et al.* [47] frequently observed more than 10^5^ clonotypes before estimating the number of unseen clonotypes. PCR amplification overestimates the repeated observation of TCR clonotype in the sample, leading to false saturation and substantial underestimates of TCR diversity. (Online version in colour.)

The principle that exhaustive sequencing does not capture full repertoire diversity was demonstrated by Warren *et al.* [52]. The authors exhaustively sequenced a library derived from a peripheral blood sample. However, upon sequencing a second library derived from the same blood sample, they found that 75% of the sequences returned were new, i.e. not contained in the first library. Furthermore, sequencing data were obtained from a second independent blood sample, and only 13% of the clonotypes observed in the second sample were observed in the first. This indicates that exhaustive sequencing of a single sample is incapable of capturing diversity, regardless of the apparent degree of repetition of species provided. That is, a saturating relationship between the number of reads and the number of clonotypes does not imply that there is a saturating relationship between the number of T cells and the number of clonotypes. The limiting factor is the number of TCRs present in the sample, not the extent of amplification or depth of sequencing. We have observed similar effects of ‘false repetition’ and ‘false saturation’ in our work [53]: figure 2 shows apparent saturation of the number of new clonotypes observed as the number of sequence reads increases. However, the number of clonotypes in the full data (2.5 × 10^4^) is a drastic underestimate of TCR diversity, where between 10^5^ and 10^6^ distinct CDR3 sequences have been directly observed [47,52]. Finally, it has been noted [54] that exhaustive sequencing of either or both of the TCR α and β chains is insufficient to capture the full repertoire of a person.

## 6. Absolute T-cell counts are required for diversity estimation

Recent approaches have used DNA barcoding [29,50,51,55] or amplicon length discrimination [56,57] to resolve the problems of PCR amplification. Under DNA barcoding, a clonotype is identified by its nucleotide or amino acid sequence, but a second identifier is assigned to each individual short DNA sequence through the addition of a random DNA sequence label. Thus, the combination of a given clonotype nucleotide sequence and a given random label is unique. This allows identical T cells to be distinguished from identical sequence reads, and so preferential amplification is irrelevant. For example, if there are two amplicons that have identical CDR3 sequences and identical labels, it can be concluded that both amplicons have been derived from a single DNA sequence. The resulting data therefore consist of absolute—not relative—clonotype abundances, which are required for any abundance-based estimator. Furthermore, DNA barcoding can be extended to correct for sequencing error [50,55].

Another factor that prevents absolute quantification of TCR abundance is the sequencing of cDNA rather than genomic DNA, since a single T cell may express multiple mRNA copies. Therefore, cDNA is not suitable for diversity estimation.

## 7. Unseen clonotypes: the problem

Even where data collection involves considerable sequencing depth, and where unbiased data have been obtained, estimators of the number of unseen clonotypes will need to be employed because of limits on blood volume that can be taken from donors. Several estimators of species richness (i.e. the number of species) developed in ecology have been applied to estimate TCR diversity, treating each clonotype as a ‘species'. Such estimators fall into two broad categories: parametric estimators [58], where the shape of the species frequency distribution is assumed to follow some analytical form, and non-parametric estimators that make no such assumptions, and thus population frequencies cannot be inferred [59]. Since the true numbers of species or clonotypes are unknown, it is difficult to validate estimators of diversity, and so in common with ecological populations, it is often unclear which estimator should be used. We compare diversity estimators below and in table 1.

**Table 1.** Comparison of diversity estimation approaches.

estimator	advantages	disadvantages
parametric (e.g. Poisson abundance models, Power laws)	can estimate clonotype frequency distribution	requires *a priori* assumptions on analytical form of clonotype frequency distribution lack of validation: goodness-of-fit to observed data does not confirm model accuracy
non-parametric abundance-based estimators (e.g. Chao1, ACE, capture–recapture)	no *a priori* assumptions required on analytical form of clonotype frequency distribution	cannot estimate clonotype frequency distribution biased by sample size inaccurate in highly diverse immunological populations
non-parametric incidence-based estimators (e.g. Chao2, ICE)	does not require absolute count data	lack of validation in immunological populations biased by sample size
DivE	accurate in multiple validations, across all immunological populations tested unbiased by sample size	time consuming: multiple models must be fitted

## 8. Differences between ecological and immunological data

There are important qualitative differences between T-cell repertoires and ecological populations in the uniformity of sampling. In ecological populations, data collection is frequently not random. For example, while placement of quadrats may be random, all of the individuals present in that quadrat are counted, leading to clustering of data [60]. Also, the probability of detection varies between species as it is influenced by colour, physical size, noise emission, geographical distribution, movement, variety of habitats and relationship to other species [61,62]. By contrast, in samples of T cells derived from blood, it is reasonable to assume that individual T cells have the same probability of detection; this assumption is less justifiable in solid tissue, as for example, lesions are non-randomly sampled.

In many ecological populations (e.g. plants, arthropods), the actual counting of individuals present in the sample is more straightforward than for populations of T cells, where sequencing introduces biases [19] and where it is difficult to distinguish sequencing errors from rare species [52]. The frequent implicit assumption that sequencing data comprised individuals that are equally detectable is often inappropriate. The probability that a given sequence read is recorded is conditional on two events: first, the probability that the T cell is sampled from blood, which is equal among T cells; and second, the probability that an amplicon from a T cell is amplified, which is not equal across all CDR3 sequences. This problem does not arise in ecological datasets.

The use of diversity indices developed in ecology that are used in T-cell repertoires is not restricted to species richness estimators. Similarity indices such as the Jaccard [63,64], Morisita-Horn [41,63], analysis of similarity (ANOSIM) [10] and dispersion metrics such as Simpson's diversity index [48,65], the Shannon entropy [20,66] and Renyi entropy [66] have been used to compare the TCR diversities between different people or between different T-cell phenotypes [65,67]. Many of the difficulties that arise in applying ecological species richness estimators to T-cell repertoires also confound the measurement of the extent of dispersion or similarity between repertoires, and ecological indices should be used with caution when analysing TCR repertoires.

## 9. Non-parametric abundance based species richness estimators

One of the most commonly used estimators is Chao1 [68] or its bias-corrected form (Chao1-bc) [69]. These estimators have been used to estimate TCR diversity in mice [70], and humans [71], making use of an amendment to the estimator [72] that takes account of the maximum upper bound of diversity.

The abundance-based coverage estimator (ACE) [73], which has been suggested as best practice [58] and is commonly used in ecology, has been used to estimate repertoire diversity in transgenic mice in the contexts of T-cell differentiation [64], and TCR specificity and self-recognition [63,74]. However, Hsieh *et al.* [74] note that ACE is based on the probability that uniform sampling would produce the observed frequency distribution.

Weinstein *et al.* [30] used a capture–recapture approach to estimate the size of the antibody repertoire in zebrafish, and this approach was extended in Glanville *et al.* [31] to estimate antibody diversity in humans. The latter study also used a technique that allows sequencing of reads long enough to span all three CDR regions, which would allow more direct data on T-cell repertoires to be collected. No validation of the capture–recapture method was performed in either study.

Non-parametric abundance-based species richness estimators have been validated using ecological populations that have been extensively sampled and where approximate species richness is assumed to be known [75]. However, the accuracy of these ecological estimators has been questioned in immunological populations. We recently compared the performance of widely used non-parametric species richness estimators (the Chao1bc [69], ACE [73], Bootstrap [76] and Good-Turing [77] estimators) from population ecology when applied to immunological and microbiological systems [53]. We considered three distinct sets of data: the clonal distribution of cells naturally infected with human T-lymphotropic virus type-1 (HTLV-1), operational taxonomic units (OTUs) of Bifidobacteria in the gastrointestinal tract of infants, and T-cell receptor repertoires. In the case of HTLV-1, a ‘species' is a clone, defined as a population of infected cells that share a genomic site of proviral integration.

**Figure 3.**
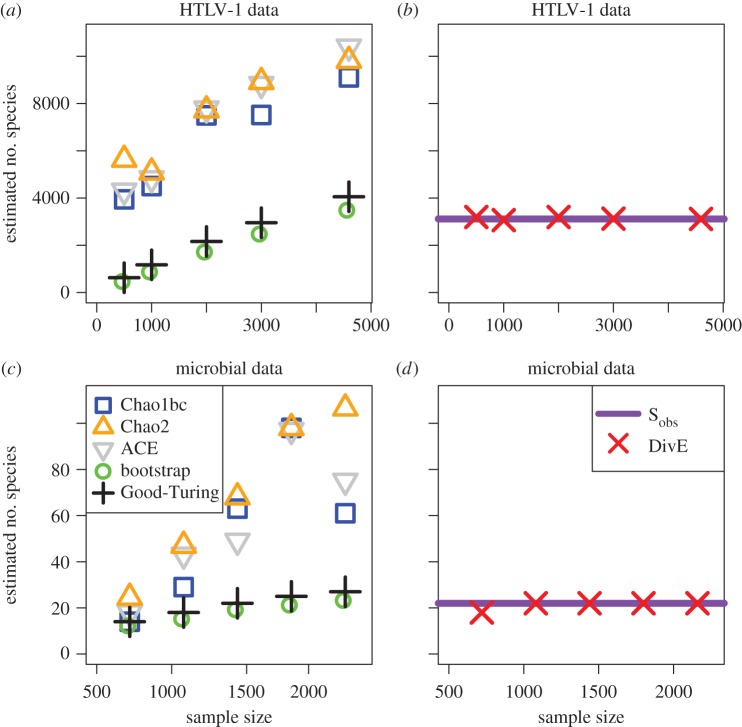
Performance of species richness estimators. (*a*,*c*) The Chao1bc (blue), Chao2 (orange), ACE (grey), Bootstrap (green) and Good-Turing (black) estimators are applied to *in silico* random subsamples of observed data. Examples for HTLV-1 and microbial data are shown. Estimates systematically increase with sample size. Chao2 estimates are calculated by randomly dividing each subsample into four *in silico* replicates. We observe the same bias with sample size where subsamples were divided into two and three *in silico* replicates (data not shown). (*b*,*d*) DivE (red) is applied to same subsamples as the other estimators. Performance of DivE was evaluated by comparing the error of estimates (*Ŝ*_obs_), to the (known) number of species *S*_obs_ in the full observed data (purple line) and by comparing estimates as a function of sample size. In all datasets, DivE accurately estimates the species richness of the full observed data from subsamples of that data and is unbiased by sample size.

**Figure 4.**
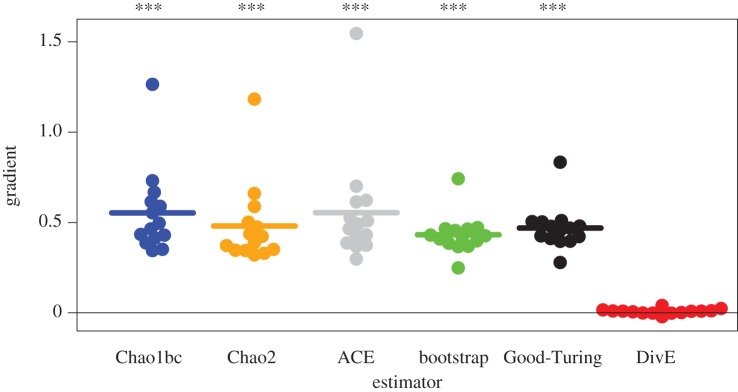
Comparison of estimators: effect of sample size on estimated HTLV-1 diversity. Gradients measuring increase in estimated HTLV-1 clonal diversity against increase in sample size. Gradients for each estimator were calculated by linear regression. All estimators except DivE show large gradients that are significantly positive, indicating a bias with sample size. ***p < 0.0001; two-tailed binomial test (*n* = 14).

For each set of data, we found that all estimators were biased by sample size (figures 3 and 4). This is problematic as estimates of species richness would increase if, for example, greater blood volumes were drawn or technique sensitivity was improved. Furthermore, there was strong evidence that the estimators underestimated diversity. Firstly, the estimators frequently produced estimates from subsamples that were lower than the diversity of the full observed sample. Secondly, in almost all cases, only a small number of unseen ‘species' was predicted in addition to those observed. Such estimates are implausible in the HTLV-1 and T-cell repertoire datasets where there is such a vast potential diversity.

## 10. Non-parametric incidence-based species richness estimators

The incidence-based coverage estimator [78] was used to estimate the diversity of regulatory T cells in transgenic mice [64], although no validation of this estimator was performed. Qi *et al.* [19] used multiple replicate libraries to compute the Chao2 estimator [79], resulting in an estimate of 10^8^ clonotypes. The Chao2 estimator makes use of incidence (i.e. presence or absence) data, as opposed to abundance data, across different replicates. The estimator therefore provides a method of avoiding the distorted abundances due to PCR amplification mentioned above.

To validate their approach, the authors created an *in silico* Zipf distribution of clonotype frequencies from which to sample. They took random samples of varying sizes and found that the estimated diversity accurately estimated the number of clonotypes in their *in silico* distribution. Although indirect and using only one *in silico* distribution, this validation suggests that their method holds promise. However, we have applied Chao2 to HTLV-1 and microbial OTU data, and we again observed a bias with sample size, as seen with the other non-parametric estimators we tested (figures 3 and 4).

## 11. Parametric species richness estimators

Robins *et al.* [47] frequently observed as many as approximately 10^5^ TCR clonotypes using single-molecule DNA sequencing, and employed a method originally devised by Efron & Thisted [80] and amended in Ionita-Laza *et al.* [81] to estimate a peripheral blood diversity of 3 to 4 × 10^6^ clonotypes, including 1 × 10^6^ antigen-experienced T-cell clonotypes, where the latter is approximately one order of magnitude higher than estimated previously [36]. Their method assumes that individual T-cell clonotypes enter the sample according to a Poisson process with clonotype-specific rates, which are inferred from the observed clonotype abundances. The method predicts the number of new sequences that would be observed in a subsequent sample. Hence their method does not merely provide an estimate of TCR diversity, but also the relationship between sample size and diversity. Therefore, the authors were able to validate their method. While this validation was direct, in that observation was compared with the predicted number of additional clonotypes, it was limited to only a single additional sample.

Several recent studies have made use of the class of Poisson abundance models (PAMs). Sepúlveda *et al.* [59] noted that species frequency data come from a multivariate hypergeometric distribution (i.e. a multinomial distribution where samples are taken without replacement). Because the size of a sample is dwarfed by the size of the total population (and therefore sampling does not drastically alter clonotype relative abundances), these authors approximated the multivariate hypergeometric distribution using a Poisson distribution. Incorporation of a varying sampling rate for clonotypes of varying frequencies leads to the class of PAMs [41,82]. They applied their method to previously published data on mice with different phenotypes, and evaluated the consistency of their method by excluding clonotypes above successively higher cut-off frequencies. Worryingly, there was wide variation in diversity estimates across all phenotypes depending on the specific PAM used. Rempala *et al.* [41] focused on one such model, the bivariate Poisson-lognormal distribution, and concluded that under-sampling in their repertoire datasets is more severe (and thus the population is more diverse) than would be estimated using the Good-Turing estimator [77]. Other extensions of the class of PAMs have been developed [41,82] that estimate the similarity between populations in the presence of unseen clonotypes.

Using a compound Poisson process model used originally to estimate gene capture diversity [83], Wang *et al.* [39] estimated TCR diversity in the context of T-cell fate and differentiation. This method can also model the relationship between the number of clonotypes and the number of T cells. They estimated approximately 10^6^ unique TCRα and TCRβ CDR3 nucleotide sequences, approximately one third of that predicted by Arstila *et al.* [36]. Their method was validated previously in the context of gene capture diversity [83] using *in silico* distributions, choosing lognormal, exponential and gamma distributions of varying diversities. It is unclear how this validation translates to T-cell immunology.

In addition to the capture–recapture approach used in Weinstein *et al.* [30], Klarenbeek *et al.* [37] fitted multiple Poisson mixture models to HTS data to estimate β-chain diversity in the CD4^+^ and CD8^+^ T-cell compartments. Extending the distribution fitted to the observed data to model the number of unseen clonotypes, the authors estimated that the memory compartment consists mainly of unexpanded clones and is far more diverse than thought previously [36] (only 2 and 3–10 times less diverse than the naive repertoire in CD4^+^ and CD8^+^ T-cell compartments, respectively). Their estimates are also remarkable in that they predict that more than 90% of memory clonotypes are relatively small.

Power laws have been used to model the form of the T-cell repertoire [84,85]. An advantage of this method is that the fitted parameters are relatively easy to interpret. It can be shown that one parameter quantifies the proportion of the repertoire occupied by clonotypes of a single T cell, and the other provides a measure of dispersion. Power law characterizations of the T-cell repertoire could be extended to estimate the number of unseen clonotypes in a similar manner to Klarenbeek *et al.* [37] by extending the modelled distribution.

Parametric approaches are often evaluated using goodness of fit to the observed data, for example using χ^2^-tests or Akaike's information criterion (AIC_c_) [30,59,86,87]. While these methods are useful for comparative purposes, they do not validate the resulting model's accuracy. A major limitation of all parametric approaches is that the estimated diversity is dependent on the assumed form of the clonotype distribution.

## 12. A new approach to T-cell receptor diversity estimation: DivE

**Figure 5.**
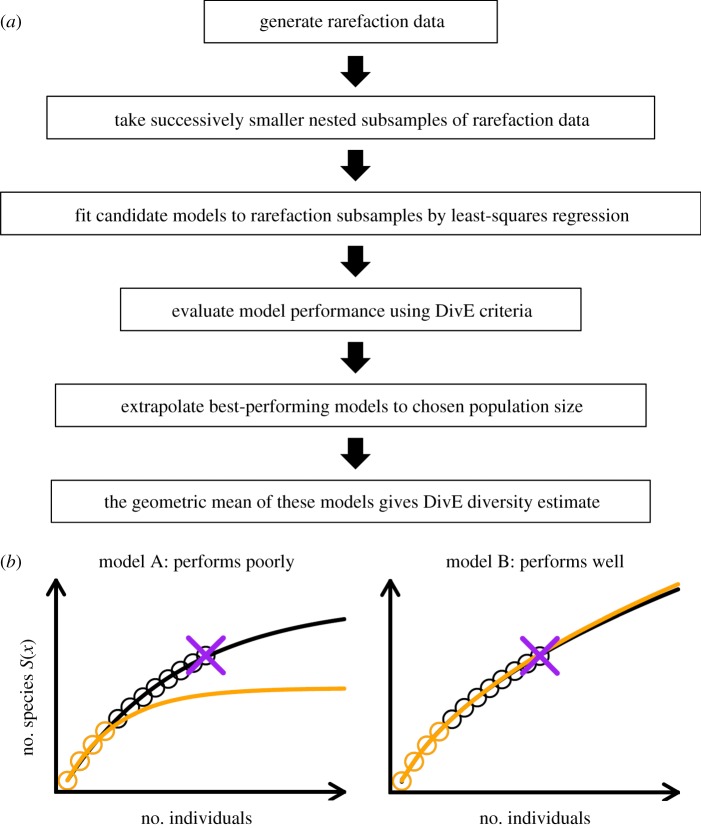
Outline of DivE species richness estimator. (*a*) Flow chart describing the process to calculate the DivE species richness estimate. (*b*) Full rarefaction curves shown in black and nested rarefaction subsample shown in orange. Data are denoted by circles, model fits by solid lines. Models are scored according to the following criteria: (i) discrepancy—mean percentage error between data points and model prediction; (ii) accuracy—error between full sample species richness (purple cross) and estimated species richness from subsample; (iii) similarity—area between subsample fit (orange) and full data fit (black) and (iv) plausibility—we require that *S′*(*x*) ≥ 0 and *S*″(*x*) ≤ 0. Model A performs poorly as criteria (ii) and (iii) are not satisfied. Model B performs well as all criteria are satisfied.

We developed an estimator named DivE [53] which uses rarefaction curves (figure 5). Similar to a species accumulation curve, an individual-based rarefaction curve is created by cumulating the number of species as the number of observed individuals (e.g. a T cell) increases, in a single resample. Species counts are averaged over multiple resamples of the data to obtain the expected number of species as a function of the number of individuals. Sample-based rarefaction curves plot the expected number of species against the number of samples.

DivE involves fitting multiple simple mathematical models, many of which are well known in ecological studies [88,89], to rarefaction curves, and to nested subsamples of these curves. Novel criteria are then used to determine the most appropriate model; as well as assessing the quality of fit to seen data these criteria also assess the quality of fit to unseen data, i.e. how well a given model can predict the full dataset from random subsets thereof. The best-performing models are then aggregated and extrapolated to a user-specified population size to produce the diversity estimate (figure 5).

We used three methods to validate the performance of DivE. We measured the extent to which DivE could: (i) estimate the diversity of the observed dataset from subsamples; (ii) estimate from a single dataset the diversity of additional independent HTLV-1 data, obtained using separate blood samples taken in immediate succession and (iii) provide consistent estimates given samples of unequal size. In each validation, the estimator performed better than the non-parametric abundance-based estimators we tested (figure 3). We believe the principal reason that DivE performs well is that candidate models are selected on their ability to consistently predict additional rarefaction data. The additional data (i.e. the full rarefaction curve) have no influence on fitted parameter values, and so DivE not only assesses goodness of fit but also evaluates the accuracy of the model. DivE has been provided as an R package [90], available at http://cran.r-project.org/web/packages/DivE/index.html [91].

Accurate extrapolation of rarefaction curves assumes that the sampled population is representative of the whole population to be extrapolated to [60,92,93]. This is a reasonable assumption in the case of T-cell sampling in the blood, i.e. T cells sampled in one blood draw are likely to be representative of all T cells in the peripheral blood. However, this is a poor assumption when trying to infer the TCR diversity in the whole body as T cells sampled in the blood may not be representative of T cells in lymphoid tissue, etc. The difficulty of inferring total population diversity from estimates in the blood is not unique to DivE and will adversely affect the accuracy of all estimators.

An alternative approach to rarefaction curve extrapolation that is based on a rigorous statistical footing has recently been developed [94–96]. However, to estimate the rarefaction curve, this method requires an input of species richness (usually provided by ACE or Chao1), which is the quantity we seek to estimate. Furthermore, the authors of these papers caution that this method is not suitable for extrapolation beyond two- or threefold.

We summarize the advantages and disadvantages of different diversity estimation methods in T-cell repertoire analysis in table 1.

## 13. Discussion

To estimate repertoire diversity it is essential to obtain unbiased data, with absolute counts of TCR clonotypes. If unbiased absolute count data are not available, neither relative abundances nor the degree of repetition of observations are credible, and so diversity estimators should not be applied. While Chao2 does not require abundance data, we have found that this estimator too is biased by sample size in immunological and microbiological data (figure 3).

We also caution against estimating diversity using severely under-sampled data, whether due to limited sequencing depth or low blood volume. To quantify ‘under-sampling’, we previously defined a parameter based on the curvature of the observed rarefaction curve (see [53] for further details). A linear rarefaction curve implies an implausible constant rate of species accumulation. As sampling depth increases, the rate of species accumulation should decrease as previously encountered species are repeatedly observed. Abundance-based estimators should not be applied when the rarefaction curve is close to linear.

Recent advances in HTS combined with DNA barcoding mean that unbiased absolute count data is now increasingly available. However, because of the enormous potential diversity of the TCR repertoire and the limited amount of blood that can be drawn from a donor at any given time, there will almost certainly be unseen TCR clonotypes regardless of the precision of data collection. Therefore, estimators of diversity must be employed. Existing parametric estimators suffer from the requirement of an *a priori* form of the species frequency distribution. Furthermore, each non-parametric estimator we have tested, either abundance- or incidence-based, was significantly biased by sample size.

Absolute count data allow important simplifying assumptions to be made about the relationship between the observed data and the underlying T-cell repertoire, namely that individual T cells have been sampled independently, randomly and with equal detection probabilities. These assumptions in turn allow the extrapolation of models fitted to individual-based rarefaction curves. The question of which model to fit, however, is non-trivial. DivE selects which models are most appropriate based on their ability to faithfully reproduce all observed rarefaction data from subsamples, providing a degree of robustness that we have not observed with classical non-parametric estimators. Crucially, the form of the model chosen depends on the data, and so DivE does not require *a priori* assumptions regarding the form of the clonotype frequency distribution, or regarding the relationship between the number of T cells and the number of TCR clonotypes.

We have validated DivE across three independent immunological and microbiological systems. In all systems, the estimator was accurate, and considerably more so than the non-parametric estimators we examined. We believe that this estimator will be an important tool to estimate T-cell repertoire diversity.
